# Supplementary material for: Genetic nurture: estimating the direct genetic effects of pediatric anthropometric traits
Source: Hum Mol Genet. 2025 Aug 21;34(20):1744–52. doi: 10.1093/hmg/ddaf117 (PMC12498280; doi:10.1093/hmg/ddaf117)
Supplement: Supplementary_Data_MCM_ddaf117 [file supplementary_data_mcm_ddaf117.docx]

Supplementary Data


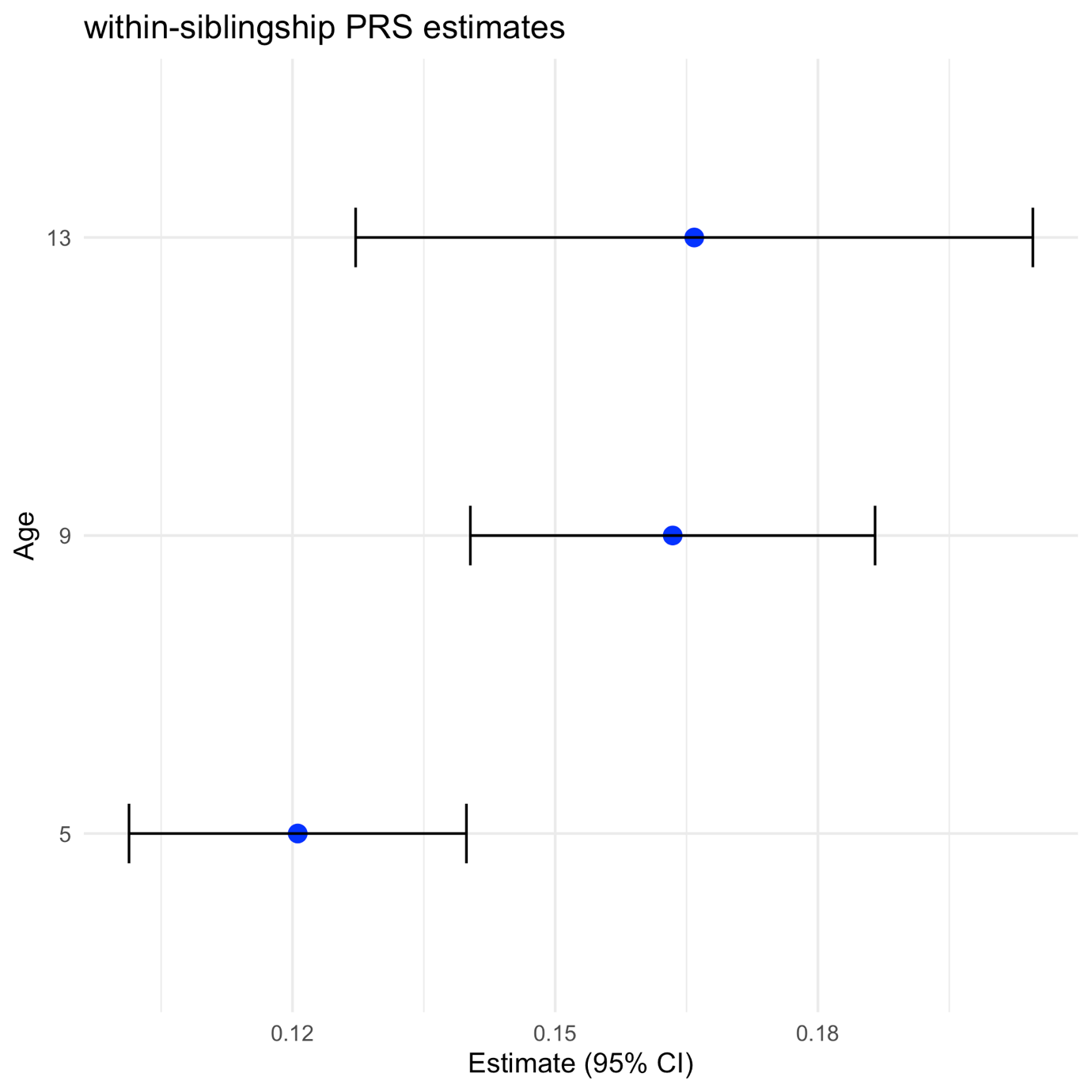


Supplementary Figure 1 -Forest plots depicting the association between PRS constructed from within-sibling GWAS for BMI, eBMD, and height with their respective traits.


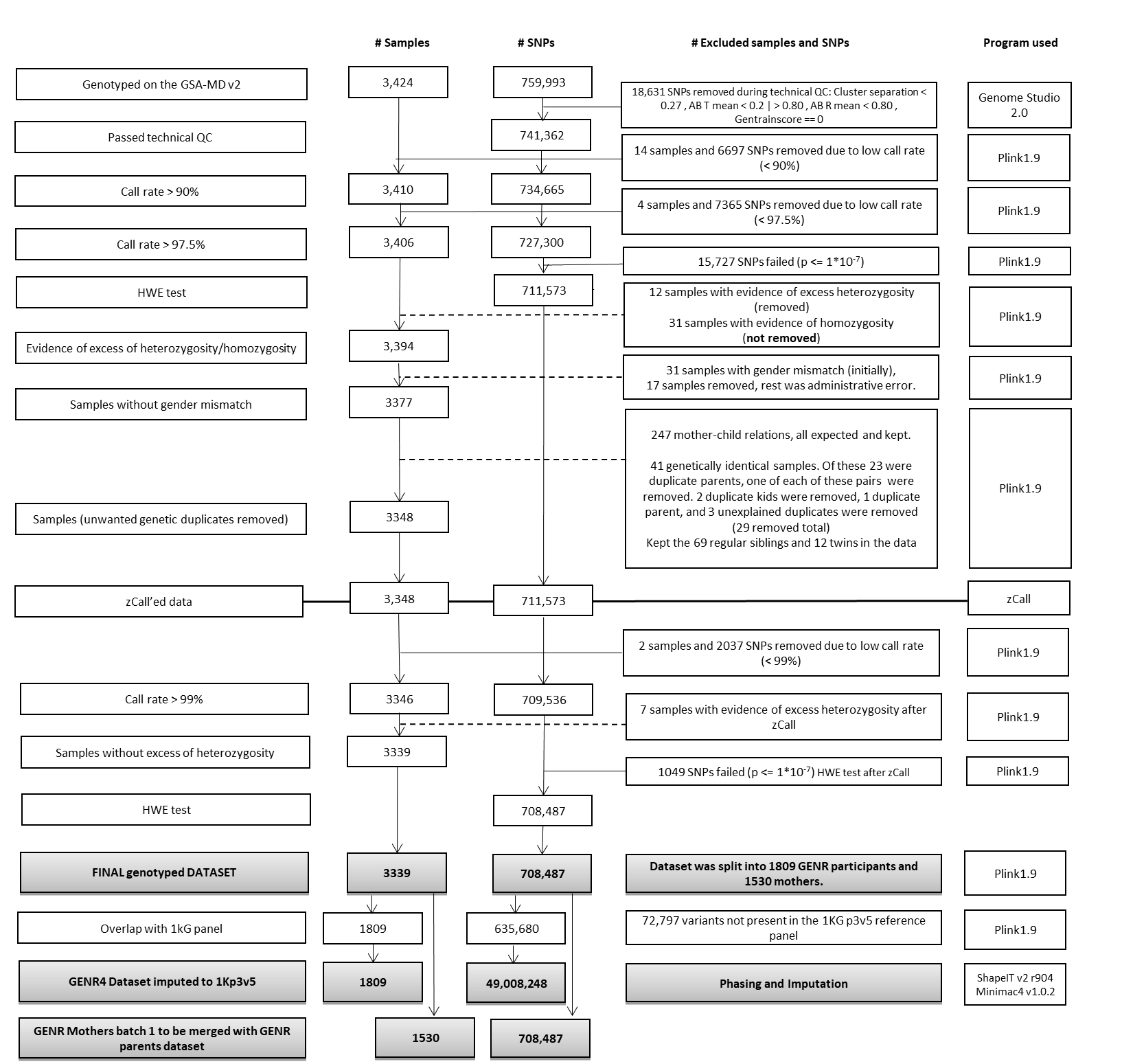


Supplementary Figure 2 – Flowchart of the quality control (QC) pipeline of the Generation R 4 and a subset of mothers genotyped with GSA-MD v2.0. The number of samples and variants retained after each QC step and imputation.


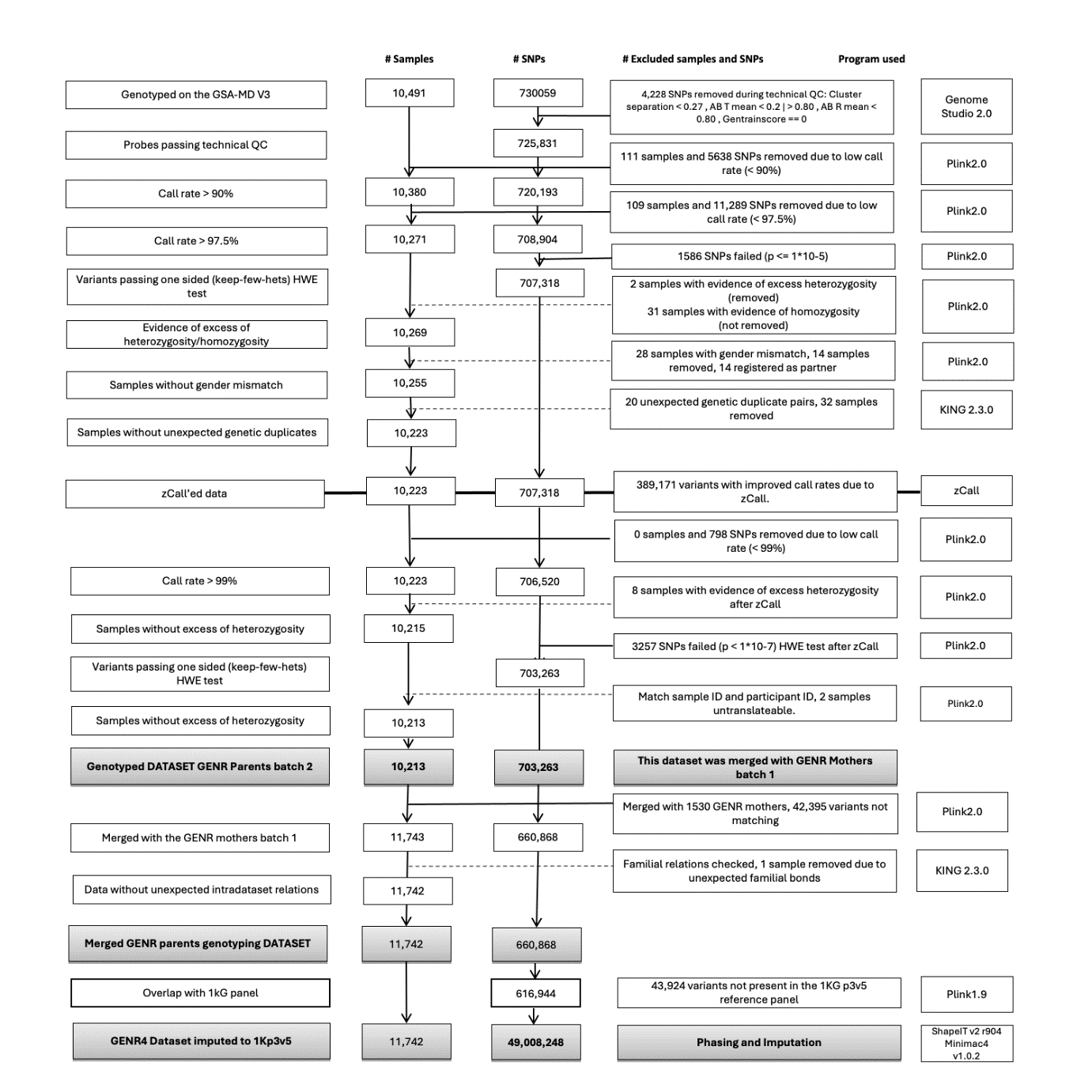


Supplementary Figure 3 – Flowchart of the quality control (QC) pipeline of the parental batch2 and merged datasets. The number of samples and variants retained after each QC step and imputation.


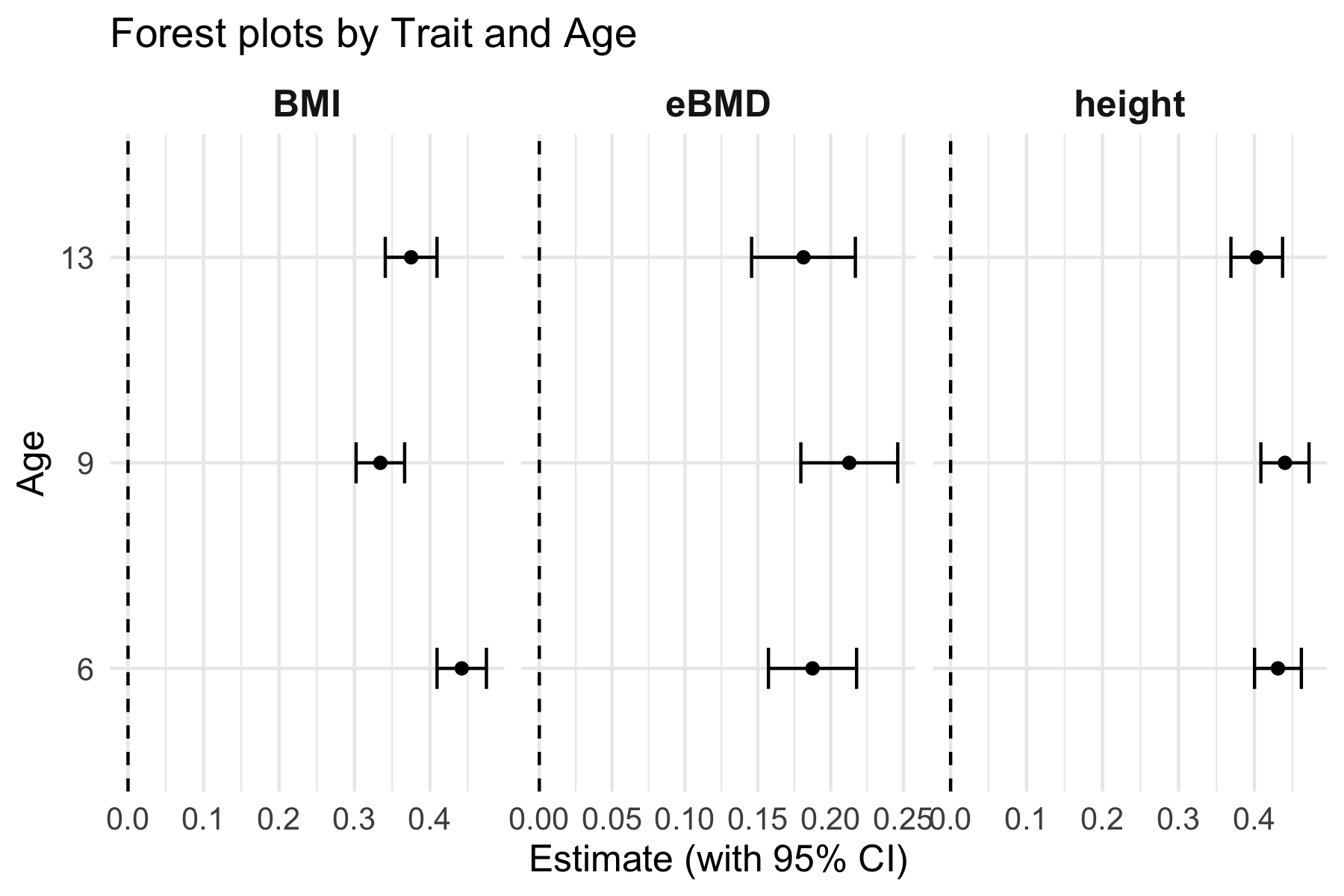


Supplementary Figure 4 – Forest plots indicating the association of BMI, eBMD, and height PRSs, constructed from European GWAS, with their respective traits across ages 13, 9, and 6.
